# When This Old Ring Was New

**Published:** 1863-10

**Authors:** G. J. Bennett


### "WHEN THIS OLD RING WAS NEW."

BY G. J. BENNETT.

Your wedding-ring wears thin, dear wife; ah! summers not a few,
Since I put it on your finger first, have passed o'er me and you;
And, love, what changes we have seen—what cares and pleasures too—
Since you became my own dear wife, when this old ring was new.

Oh! blessings on that happy day, the happiest of my life,
When, thanks to God, your low sweet "Yea," made you my loving wife;
Your heart will say the same, I know; that day's as dear to you,
That day that made me yours, dear wife, when this old ring was new!

How well do I remember now, your young sweet face that day;
How fair you were—how dear you were—my tongue could hardly say;
Nor how I doated on you; ah! how proud I was of you;
But did I love you more than now, when this old ring was new?

No—no; no fairer were you then, than at this hour to me,
And dear as life to me this day, how could you dearer be!
As sweet your face might be that day as now it is, 'tis true,
But did I know your heart as well, when this old ring was new?

O partner of my gladness, wife! what care, what grief is there,
For me you would not bravely face—with me you would not share?
Oh! what a weary want had every day, if wanting you,
Wanting the love that God made mine when this old ring was new!

Years bring fresh links to bind us, wife—small voices that are here,
Small faces round our fire that make their mother's yet more dear,
Small, loving hearts, your care each day makes yet more like to you,
More like the loving heart made mine when this old ring was new.

And, blessed be God, all he has given are with us yet; around
Our table, every little life lent to us, still is found;
Though cares we've known, with hopeful hearts the worst we've struggled through;
Blessed be His name for all his love since this old ring was new.

The past is dear; its sweetness still our memories treasure yet;
The griefs we've borne, together borne, we would not now forget;
Whatever, wife, the future brings, heart unto heart still true,
We'll share as we have shared all else since this old ring was new.

And if God spare us 'mongst our sons and daughters to grow old,
We know his goodness will not let your heart or mine grow cold.
Your aged eyes will see in mine all they've still shown to you,
And mine in yours all they have seen since this old ring was new.

And oh! when death shall come at last to bid me to my rest,
May I die looking in those eyes, and resting on that breast;
Oh! may my parting gaze be blessed with the dear sight of you,
Of those fond eyes—fond as they were when this old ring was new.

